# The combination of nasal steroids and anti-leukotriene to reduce adenectomy in children with OSA and adenoid hypertrophy

**DOI:** 10.5339/qmj.2023.sqac.31

**Published:** 2023-11-26

**Authors:** Manal Eldegeir, Noorah AL Marry, Fatima Awami, Fatima Alsada

**Affiliations:** ^1^National Guard Dammam, Dammam, KSA Email: almanjil@gmail.com

## Abstract

Background: Obstructive sleep apnea (OSA) affects 1% to 5% of all children, with the most significant prevalence between ages 2 and 8. Correlations between OSA and Adenoid hypertrophy (AH) have been well-demonstrated in children. If untreated, OSA can cause growth impairment, neuro-cognitive and behavioral problems, and cardiovascular complications. Allergy was reported to be a vital risk factor for AH, among other inflammatory processes mentioned.

Objectives:

1. To demonstrate that the combination of nasal steroids and ani leukotriene reduces the number of adenectomies in children with OSA and adenoid hypertrophy in Saudi children.
2. To demonstrate the positive Correlation between allergic sensitization and OSA caused by adenoid hypertrophy

Methods: This retrospective study included 60 children with moderate/severe OSA attending a Pediatric Allergy Clinic in Saudi Arabia diagnosed using Sleep-Related Breathing Disorder Scale (pediatrics-related items). We had children with adenoid hypertrophy confirmed by soft neck tissue x-ray or both; children who suffer from obesity were excluded. Sensitization was defined as positive specific IgE and/or skin prick for food and/or inhaled allergens; allergic comorbidities were enumerated.

Results: Combination of nasal steroid and anti-leukotriene reduces adenectomy by 58.3 % in children with OSA caused by AH. 71.7% of Them were sensitized to inhaled food or both allergens.

Conclusion: Anti-inflammatory treatment with a combination of nasal steroids and anti-leukotriene remarkably reduces the adenectomy rate in children with OSA caused by AH. Our findings suggest that allergic sensitization, regardless of the allergen, increases children’s susceptibility to AH through the inflammatory process. This may be why nasonex and montelukast are effective treatments for OSA in children with AH.

